# Clinical Effect of Revascularization Strategies and Pharmacologic Treatment on Long-Term Results in Patients with Advanced Peripheral Artery Disease with TASC C and D Femoropopliteal Lesions

**DOI:** 10.1155/2022/3741967

**Published:** 2022-03-04

**Authors:** Chiu-Yang Lee

**Affiliations:** ^1^Division of Cardiovascular Surgery, Department of Surgery, Taipei Veterans General Hospital, Taipei, Taiwan; ^2^Institute of Clinical Medicine, National Yang Ming Chiao Tung University, Taipei, Taiwan; ^3^National Defense Medical Center, Taipei City, Taiwan

## Abstract

**Background:**

This study was to assess the clinical outcome and associated parameters of endovascular therapy (EVT group) and bypass surgery (bypass group) in patients with long femoropopliteal TransAtlantic Inter-Society Consensus II (TASC II) C and D peripheral artery disease (PAD).

**Methods:**

187 patients who underwent successful EVT or bypass surgery were assessed. The endpoints included the events of cardiovascular disease (CVD) and lower-extremity amputation (LEA), 3-year primary patency, and 3-year amputation-free survival (AFS).

**Results:**

The 3-year primary and secondary patency rates were better in the bypass group (*P*=0.007 and *P*=0.039, respectively), while the incidences of LEA, new CVD events, and mortality were comparable between groups. Weighted multivariate Cox analyses showed that cilostazol treatment (hazard ratio (HR): 0.46, 95% confidence interval (CI): 0.3–0.72, *P*=0.001), statin treatment (HR: 0.54, 95% CI: 0.33–0.9, *P*=0.014), and direct revascularization (DR) (HR: 0.47, 95% CI: 0.29–0.74, *P*=0.001) were predictive factors of 3-year primary patency. Kaplan–Meier curve analyses of time-to-primary cumulative AFS showed that nondiabetes mellitus, mild PAD, and cilostazol and statin treatment were correlated with a superior 3-year AFS (log rank test, *P*=0.001, *P* < 0.001, *P*=0.009, and *P*=0.044, respectively).

**Conclusions:**

Endovascular stenting based on the angiosome concept and bypass surgery provide comparable benefits for the treatment of long, advanced femoropopliteal lesions after a short follow-up period, whereas cilostazol therapy for more than 3 months, aggressive treatment of dyslipidemia, and surgical revascularization were associated with higher primary patency.

## 1. Introduction

Peripheral arterial disease (PAD) is a prevalent type of atherosclerosis and is similar to coronary artery disease (CAD), which is also caused by atherosclerosis.

Critical limb ischemia (CLI) is associated with high cardiovascular disease mortality and lower-extremity amputation (LEA) [1, 2]; it is the most severe form of PAD, presenting as gangrene, pain at rest, and ischemic ulcer necrosis. The TransAtlantic Inter-Society Consensus (TASC) II guidelines recommend revascularization approaches including endovascular intervention and bypass surgery for CLI. Recent series of studies have shown that 10–20% of lower-limb revascularization procedures are performed surgically, and approximately 80% are endovascular. The TASC II guidelines recommend endovascular intervention as the optimal option for the treatment of CLI to relieve pain, assist wound healing, prevent limb loss, and improve patient function and quality of life.

Despite the advantages of endovascular intervention for CLI management, including a lower surgical risk and lower mortality, mean patency rates of 56% to 77% at 1 year and 39% to 80% at 5 years have been reported, depending on the revascularization method and location of the disease [3–5]. Taking long-term durability into consideration, bypass surgery remains the optimal treatment for multilevel and long femoropopliteal lesions not subject to endovascular intervention and provides adequate arterial perfusion to the foot, resulting in an elevated limb salvage rate and long-term durability [6, 7]. The aforementioned studies demonstrated that in the first year, approximately 25% of these patients experienced resolution of their symptoms, while 20% continued to have symptoms, 30% underwent amputation, and 25% expired. Taking the results of all of the aforementioned reviews together, the unique emphasis on surgical or endovascular revascularization strategies as the basis of current treatment for long femoropopliteal lesions in patients with PAD was not sufficient without comprehensive strategies for reduction of restenosis and arterial remolding.

To our knowledge, evidence regarding the presence of cardiovascular risk factors, morbidity, and mortality associated with revascularization methods has been presented, but data regarding the effects of individual predisposing factors and pharmacotherapy on the primary patency of target lesion, clinical outcomes, and limb function are conflicting. The aim of the study was to investigate the clinical outcome and associated parameters in patients with CLI receiving endovascular therapy and bypass surgery.

## 2. Materials and Methods

### 2.1. Study Population and Design

The study was conducted in accordance with the Declaration of Helsinki. Prior to the study, the protocol was approved by the Ethics Committee of Taipei Veterans General Hospital (No. 2015-03-016AC). All patients gave written informed consent before revascularization. This study retrospectively analyzed our prospectively maintained institutional database, which included 595 consecutive advanced PAD patients (Rutherford classification III, IV, V, and VI) who underwent bypass surgery or endovascular therapy (EVT, angioplasty plus bare metal stent (BMS) or covered stent (Viabahn)) in a single medical center from February 2009 to March 2015. Of these, 187 patients with 229 limbs satisfied the inclusion criteria discussed further.

The inclusion criteria were patients between the ages of 20 and 90 years; with a first diagnosis of PAD; severe PAD (Rutherford classification III, IV, and V) manifested as severe claudication, rest pain, or ischemic tissue loss; ischemic tissue loss associated with an ankle pressure <70 mmHg or a toe pressure <50 mmHg; long femoropopliteal TASC C and D lesions; successful bypass surgery or endovascular therapy (stenting), obtaining flow through at least one vessel to the pedal arch; treatment with cilostazol; and fair compliance and regular monitoring during follow-up. The exclusion criteria included acute artery embolism; Buerger's disease; prior amputation (minor or major); congestive heart failure (CHF) according to New York Heart Association (NYHA) function III–IV or an ejection fraction of <35%; bleeding diathesis, acute cardiovascular diseases, or acute cerebrovascular diseases; and active diseases such as hepatitis, malignancy, or systemic infection. These patients were excluded before intervention.

According to the angiosome-oriented revascularization strategy, endovascular and surgical revascularization were applied in this study. If patients had plantar ulcers, we made an effort to treat the posterior tibial artery first. If this artery was not revascularized, we then treated the anterior tibial artery. Direct revascularization (DR) provides adequate blood flow directly to the ischemic tissue area over the direct angiosome, while indirect revascularization (IR) provides blood flow to the ischemic limb over the indirect angiosome through the collateral vessels [8–10].

In total, 187 patients with advanced PAD mainly involving long femoropopliteal lesions (TASC II C and D) were included in this study. The patients were divided into bypass (bypass surgery) and EVT groups.

### 2.2. Medication

Patients undergoing stenting were given dual antiplatelet therapy for at least 3 months followed by single antiplatelet therapy. For patients undergoing bypass surgery, single antiplatelet therapy was prescribed. All patients in this study were treated with cilostazol (50–100 mg bid) after the intervention. Patients who received continuous cilostazol therapy for at least 3 months after surgery were considered to have received long-term cilostazol therapy, while those who received intermittent treatment or treatment for fewer than 3 months were defined as receiving short-term treatment. The duration of cilostazol treatment depended on resolution of the claudication and leg pain, and relief of the clinical condition. In addition, fewer than half of the patients were treated with statins to control total cholesterol and LDL levels to 200 mg/dl and 130 mg/dl, respectively.

### 2.3. Study Endpoints

All patients underwent clinical, ABI, and Doppler ultrasound examinations every three to six months. CTA or invasive angiography was performed when abnormal findings occurred. The primary endpoints were the primary patency rate of endovascular intervention or graft bypass of femoropopliteal lesions at 6, 12, 24, and 36 months; and the secondary patency rate without extra endovascular stenting or graft bypass for target lesions at 12, 24, and 36 months. In this study, LEA was defined as a mild amputation (below the ankle) or a major amputation (above the ankle). The secondary endpoints were amputation-free survival (AFS), overall survival (OS), or overall mortality associated with a cerebrovascular accident (CVA) or sepsis at 24 months, and CAD at 12, 24, and 36 months. AFS in this study was defined as the avoidance of major amputations.

Successful revascularization was defined as residual stenosis of less than 30% upon angiography, with an indication of a minimum of one BTK artery or good distal collateralization to the plantar arch, or a postprocedural increase in the ABI by 0.10 or in ankle pressure as compared with baseline measurements.

A major adverse limb event (MALE) based on the Society for Vascular Surgery (SVS) document is a fundamental outcome measure for comparison of revascularization approaches, including target lesion reintervention (thrombectomy/thrombolysis or major surgical revision of an existing bypass or new stenting/open bypass graft) in addition to major amputation.

Target lesion reintervention encompasses endovascular or graft bypass procedures for occlusive lesions, performed in patients with recurrent symptoms accompanied by a decrease in the ABI by 0.10 during follow-up or a return to the prior condition and recurrent stenosis <50% as measured by duplex ultrasound imaging or CTA, or invasive angiography results showing a stenosis diameter of ≤50% or a stenosis area of ≤70% [11, 12].

### 2.4. Statistical Analysis

The Kolmogorov–Smirnov test was employed to examine the normality of numerical variable distributions. According to the distributed results, continuous data are presented as the mean ± standard deviation (SD), and percentages and numbers are used to express categorical data. Frequencies and categorical variables were compared between groups using the *χ*^2^ test.

For numerical variables, the Mann–Whitney *U* test and the independent sample *t* test were used to determine intergroup differences. Patients were undergoing follow-up when any of the following conditions occurred: death, major amputation, or patency failure for cumulative patency. The Cox regression method was used to identify susceptibility factors for primary patency for 36 months. Additionally, these significant and potential factors with *P* values <0.15 were analyzed using a weighted multivariate Cox regression model to determine their associations with primary patency based on the average treatment effect (ATE) [13, 14]. Logistic regression was used to assess inverse probability weighting propensity scores (PSs) based on the severity and location of the disease. Inverse probability weighting PSs were determined according to disease severity and location using logistic regression to measure the ATE or the average treatment effect on the treated (ATT). A 2-tailed *P* < 0.05 was deemed significant. All analyses were performed using SPSS version 22.0 (SPSS Inc., Chicago, IL, USA).

## 3. Results

### 3.1. Baseline Characteristics

From February 2009 to March 2015, a total of 187 subjects were enrolled in this study.

The EVT group included 94 patients (26 females, 27.7%; 75.8 ± 13.4 years of age), while the bypass group included 93 patients (26 females, 28%; 74.4 ± 10.3 years of age).

The patients in the two groups were well-matched in terms of the clinical characteristics and baseline demographics associated with bypass surgery and endovascular revascularization, as shown in Table 1. All patients had advanced PAD manifested as severe claudication (9.1%), resting ischemic pain (25.7%), or tissue loss (74.3%) (Rutherford category ≥ V).

A summary of the TASC lesions, distribution of diseased vessels, and interventional characteristics is presented in Table 2. All diseased arteries were characterized using pretreatment imaging results based on the stratification of lesions as per the updated 2015 TASC II classification for aortoiliac, femoropopliteal, and infrapopliteal lesions [3]. As presented in Table 2, patients in the bypass and EVT groups had multiple lesions, and the bypass group had more advanced femoropopliteal lesions (TASC D) (<0.001). All lesions were greater than 15 cm in both groups, and the patients in the bypass group had significantly longer femoropopliteal lesions as compared with the EVT group (18.5 ± 3.6 cm vs. 23.4 ± 5.2 cm, *P* < 0.001). All patients had at least one patent distal runoff of the foot, and there were no significant differences in the distal runoff vessels between the bypass and EVT groups (1.96 ± 0.79, 1.78 ± 0.72, *P*=0.146). The patients in the EVT group required BTK intervention to create better distal perfusion to the foot without the use of stents or drug-coating balloons (DCB); 29 patients (31.2%) in the bypass group required femorodistal bypass to the BTK artery.

### 3.2. Endpoints

The associations of treatment methods with study endpoints at 36 months for all participants and subgroups are summarized in Table 3. In this study, 94 (100%) patients in the EVT group received balloon angioplasty plus bare metal stent (BMS) or covered stent (Viabahn) implantation, and 93 (100%) patients in the bypass group underwent bypass revascularization.

All enrolled patients experienced successful surgery, and no surgical mortality or major complications such as massive hematoma, retroperitoneal bleeding, or pseudoaneurysm occurred in either group.

Both groups had comparable ABI values before the intervention and one month after (*P*=0.473 and *P*=0.113, respectively), but the bypass group had a significantly higher ABI value at the 3-year follow-up point (0.53 ± 0.16 vs. 0.72 ± 0.14, *P* < 0.001).

**Figure 1:**
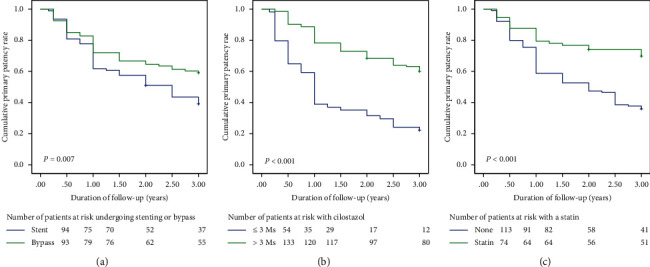
(a) Patients who underwent bypass surgery had a better patency rate than patients who received stenting treatment (log rank test, *P*=0.007). (b) Patients who received cilostazol treatment for more than 3 months had a patency rate than patients who received treatment for fewer than 3 months (log rank test, *P* < 0.001). (c) Patients receiving statin treatment had a patency rate than those who did not receive treatment (log rank test, *P* < 0.001).

**Figure 2:**
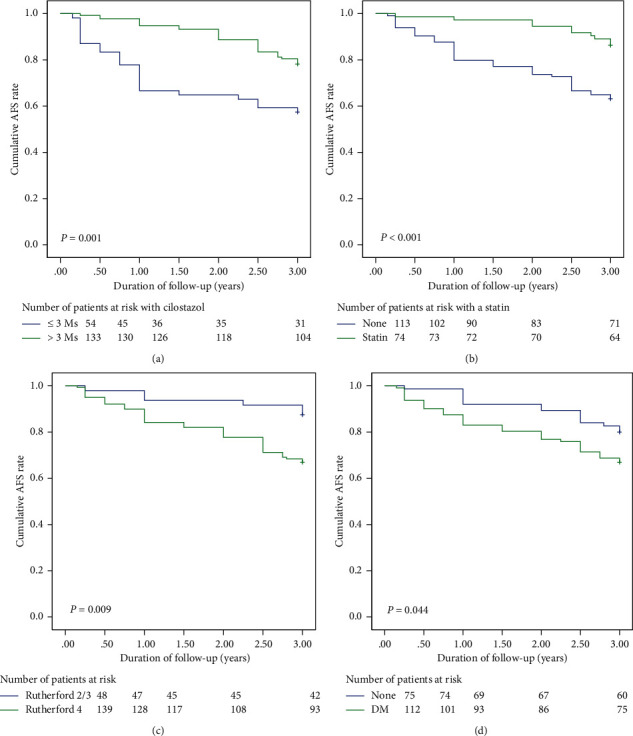
(a) Patients who received cilostazol treatment for more than 3 months had a better 3-year amputation-free survival (AFS) rate than patients who received treatment for fewer than 3 months (log rank test, *P*=0.001). (b) Patients receiving statin treatment had a superior 3-year AFS rate to those who did not receive treatment (log rank test, *P* < 0.001). (c) Patients who presented with TASC D peripheral artery disease (PAD) had an inferior 3-year AFS rate than patients who had TASC B plus C PAD (log rank test, *P*=0.009). (d) Patients with diabetes mellitus had a poorer 3-year AFS rate than patients without DM (log rank test, *P*=0.044).

The two groups had comparable percentages of patients undergoing DR (*P*=0.512). The 1- and 2-year primary patency rates of the groups were comparable, whereas the bypass group had a greater primary patency rate at 3 years as compared with the EVT group (65.6% vs. 42.6%, *P*=0.001). The secondary patency rate was similar in both groups at 24 months, and the bypass group had a better secondary patency at 3 years (*P*=0.046). The Kaplan–Meier curves employed to analyze time-to-primary cumulative patency and AFS at 3 years are presented in Figures 1 and 2. The 3-year primary patency results demonstrated the following: (A) patients who received bypass surgery had a better patency rate than patients who received stenting (log rank test, *P*=0.007); (B) patients treated with cilostazol for more than 3 months had a better survival rate than those treated for less than 3 months (log rank test, *P* < 0.001); and (C) survival rates of patients treated with statins were better than those of patients who did not receive statins (log rank test, *P* < 0.001) (Figure 1).

The 3-year AFS results showed the following: (A) patients who received cilostazol treatment for more than 3 months had a better 3-year AFS than patients who received treatment for fewer than 3 months (log rank test, *P*=0.001); (B) patients who received statin treatment had a superior 3-year AFS than those who did not receive treatment (log rank test, *P* < 0.001); (C) patients presented with Rutherford class V plus VI had an inferior 3-year AFS than patients with Rutherford class III plus IV (log rank test, *P*=0.009); and (D) patients with diabetes mellitus had a poorer 3-year AFS than patients without (log rank test, *P*=0.044).

The 3-year AFS was 68.1% (64) and 76.3% (71) in the ETV and bypass groups, respectively (*P*=0.208), while the 24- and 36-month OS rates were similar in the two groups (*P*=0.199 and *P*=0.134). Regarding new CAD and CVA events, there were no significant differences between the ETV and bypass groups at 12, 24, and 36 months. In total, 31 patients died during the 36-month follow-up period, 17 due to cardiogenic shock related to cardiovascular disease and 7 due to associated foot sepsis; three uremic patients died due to complications of major surgery, and the remaining 4 expired due to sepsis related to pneumonia.

### 3.3. Assessment of Primary Patency

In the weighted univariate Cox regression analysis of the 36-month primary patency, the significant factors were long-term cilostazol usage, statin treatment, insulin use, diabetic neuropathy, intervention (bypass/stenting), and DR. The results of multivariate Cox regression analysis showed that three main factors were independently associated with primary patency for 36 months: cilostazol treatment for more than 3 months (HR: 0.46, 95% CI: 0.3–0.72, *P*=0.001), statin treatment (HR: 0.54, 95% CI: 0.33–0.9, *P*=0.0179), and DR (HR: 0.47, 95% CI: 0.29–0.74, *P*=0.001) (Table 4).

Furthermore, in the subgroup multivariate Cox regression analysis, cilostazol treatment (HR: 0.46, 95% CI: 0.26–0.82, *P*=0.009) and DR (HR: 0.43, 95% CI: 0.22–0.81, *P*=0.009) were independent factors associated with the 36-month primary patency in the EVT group, whereas the only main factor in the bypass group was cilostazol treatment (HR: 0.33, 95% CI: 0.15–0.75, *P*=0.008) (Table 5).

## 4. Discussion

Restenosis and intimal hyperplasia are the main problems that occur after endovascular revascularization with percutaneous transluminal angioplasty or stenting. Despite the popularity and advancement of endovascular revascularization for CLI, improving the clinical outcome and preservation of limb function remain challenging following revascularization in long femoropopliteal lesions.

In this study, almost 60% of the patients had diabetes, 38% had CAD, 27.4% had hyperlipidemia, 82.4% had hypertension, and more than 90% of patients met the criteria for CLI. Most of the patients had at least two levels of complicated arterial occlusion, mainly involving femoropopliteal and infrapopliteal lesions, and partly aortoiliac lesions. In comparison with published literature regarding self-expanding polytetrafluoroethylene (ePTFE)-covered stents (Viabahn; W. L. Gore and Associates, Inc., Flagstaff, AZ, USA) and bare metal stents (BMS) [15–17], which reported the percentage of CTO lesions to be 56–70%, with 35–44% of patients having diabetes, 26–28% experiencing hyperlipidemia, 22–36% with CAD, and 14–19% experiencing tissue loss, the percentage of patients with comorbidities was higher in this study.

Numerous studies have demonstrated that a greater number of comorbid conditions and risk factors are associated with poor durability of patency, high risk of mortality, and failure of limb salvage [18–21]. In comparison with these aforementioned studies, our patient group was of a more complex composition, but the 2-year major amputation rate (7.3% vs. 8.0%), 2-year primary patency rate (63.6% vs. 56.4%), and 2-year secondary patency rate were comparable (85.5% vs. 80.9%). The main reason for the inferior 2-year primary patency rate was that the stenting instruments used in this study included BMS and Viabahn for femoropopliteal lesions, combined with balloon angioplasty for infrapopliteal lesions; Viabahn was not available in our institution prior to October 2011, and therefore BMS was the only device being used before that time. Numerous recent trials have demonstrated that BMS provides an efficient treatment for short lesions but is subject to neointimal hyperplasia and carries risks of in-stent restenosis (ISR) and stent fracture when used in long lesions, while the covered stent (Viabahn) offers more ideal outcomes in long stenotic or occlusive femoropopliteal lesions (TASC C and D lesions) and satisfactory prevention of neointimal growth, but with frequent occurrence of edge stenosis with graft thrombosis [15, 22–26].

The results of this study showed the 3-year primary and secondary patency rates of the bypass group to be 65.6% and 81.7%, as compared with several bypass studies reporting primary patency rates of 57.2–76.2% and 62.6–81.8% [27]. This study did not present a notable 3-year primary patency rate, and there was a comparable 3-year secondary patency rate because this study included patients with long, complex, and multilevel arterial lesions.

The Bypass Versus Angioplasty in Severe Ischemia of the Leg (BASIL) study demonstrated that the surgical group had a lower rate of repeated revascularization and no differences in major amputation or mortality for more than 5 years as compared with the endovascular group were noted, despite the higher risks of myocardial infarction, wound infection, and pulmonary complications [28]. Our study presented comparable 1- and 2-year primary patency rates and no differences in mortality, amputation, or cardiovascular events at the 3-year follow-up point, but the bypass group had better 3-year primary and secondary patency rates. Our finding that bypass surgery may provide a more durable patency for long SFA lesions as compared with EVT was partly concordant with the results of the BASIL study [28]. Endovascular devices and techniques have been greatly improved since the BASIL trial, and there are more strategies for maintenance of graft patency and improvement of limb salvage with endovascular intervention [29–33]. To date, endovascular revascularization has been advocated as the first option for CLI treatment, but long and complicated SFA lesions are subject to flexion, compression, and torsion close to the popliteal area, which leads to poorer durability of BMS and graft stenting, whereas surgical revascularization provides a straight-line flow into the foot, promotes wound healing, and limits the need for amputation. Accordingly, surgical bypass may be an alternative option for the treatment of long lesions in advanced PAD. Taking these results together, the optimal cure strategy (bypass surgery versus endovascular revascularization) demands a comprehensive understanding of anatomic configurations, patient conditions and preferences, surgeon experience and skill, and a multidisciplinary approach to promote a better clinical outcome and quality of life.

Evidence that pharmacologic therapies prevent restenosis or stent thrombosis after bypass surgery or endovascular therapy is sparse and often inferred from studies of coronary artery interventions regarding antiplatelet therapy. The ACC/AHA guidelines for PAD management recommend that cilostazol treatment is effective in improving intermittent claudication and increasing walking distance [34]. Clinical studies of the benefit of cilostazol treatment for improvement of patency and limb salvage are rare, and most trials suggest that cilostazol may reduce the ISR in patients with coronary intervention and those with femoropopliteal lesions [35–39].

Cilostazol is an antiplatelet drug with multiple effects, including inhibition of platelet aggregation and proliferation of smooth muscle cells, and then promotion of vasodilation and increased peripheral blood flow [40–42]. Accordingly, this preliminary study demonstrated that long-term administration of cilostazol was a significant predictor of 36-month primary patency and AFS after open surgical or endovascular revascularization. However, this study provided no direct evidence that cilostazol treatment led to improvements in wound healing or limb salvage. Further research is needed in this field.

This study also assessed whether the use of statins is associated with primary patency of target lesions. Of the 187 patients, 74 (39.6%) received postoperative treatment with statins to control total cholesterol and LDL levels. The results demonstrated that the patients under long-term statin therapy experienced great benefits in terms of the 3-year patency of target lesions and increased AFS. This result was somewhat in line with the report of the CRITISCH registry, which indicated that the use of statins in patients with CLI is associated with a better AFS and a lower LEA and mortality rate [43]. Further studies focusing on the effects of statin treatment should be carried out to clarify this issue.

Several trials have demonstrated that the outcomes in terms of wound healing and limb preservation after IR in the presence of collaterals are similar to the outcomes after DR [8–10, 44]. Our multivariate Cox regression analyses of patency revealed that DR may provide a straight-line flow to the foot and improve the primary patency at 36 months in the bypass and EVT groups. Especially, DR may be an independent predictor of 3-year primary patency after endovascular stenting. Further research may be needed in this field.

There were some limitations to our study. The first was that this study was a single-center, retrospective analysis of a prospectively-maintained database that did not have sufficient functionality for extensive statistical comparisons. Second, this was not an analysis of single femoropopliteal lesions or TASC classification, which may have led to some differences being derived from selection bias. To reduce bias, weighted multivariate Cox regression analysis using a propensity score-based approach was used to assess the associations of factors based on TASC disease severity and location with primary patency. Third, the time interval during which the patients were enrolled was long, and changes in the techniques and devices employed could have influenced the results.

## 5. Conclusions

Our study did not show any significant difference in outcome between endovascular stenting based on the angiosome concept and bypass surgery for the treatment of long, advanced femoropopliteal lesions after a short follow-up period, whereas cilostazol therapy for more than 3 months, aggressive treatment of dyslipidemia, and surgical revascularization were associated with higher primary patency. A large-scale, prospective, randomized study should be conducted for further exploration.

## Figures and Tables

**Table 1:** Baseline demographics and characteristics associated with intervention.

Characteristic	Total population (*n* = 187)	EVT group (*n* = 94)	Bypass group (*n* = 93)	*P* value
Age (years)	75.1 ± 11.9	75.8 ± 13.4	74.4 ± 10.3	0.424
BMI	23.4 ± 4.4	24 ± 3.7	22.8 ± 5.0	0.079
SBP	137.1 ± 24.1	137.1 ± 24.1	137.1 ± 24.2	0.988
Gender (female)	52 (27.8)	26 (27.7)	26 (28.0)	0.964
Lesion site (left)	91 (48.7)	45 (47.9)	46 (49.5)	0.828
Baseline CAD	71 (38)	34 (36.2)	37 (39.8)	0.611
ESRD (regular dialysis)	66 (35.6)	36 (38.3)	30 (32.3)	0.388
DM	112 (59.9)	61 (64.9)	51 (54.8)	0.161
Hypertension	154 (82.4)	76 (80.9)	78 (83.9)	0.588
Hyperlipidemia	51 (27.3)	25 (26.6)	26 (28.0)	0.321
Baseline CVA	24 (12.8)	15 (16)	9 (9.7)	0.199
Smoking	75 (40.3)	35 (37.6)	40 (43.0)	0.455
Cellulitis on presentation	145 (77.5)	74 (78.7)	71 (76.3)	0.697
Atrial fibrillation	14 (7.5)	9 (9.6)	5 (5.4)	0.275
Baseline ABI	0.52 ± 0.13	0.51 ± 0.11	0.53 ± 0.16	0.473
Calcification	75 (43.6)	36 (42.4)	39 (44.8)	0.744
Retinopathy	28 (15)	18 (19.1)	10 (10.8)	0.108
Neuropathy	49 (26.2)	29 (30.9)	20 (21.5)	0.146
Rutherford classification				0.757
Stage III	17 (9.1)	10 (10.6)	7 (7.5)	
Stage IV	41 (21.9)	20 (21.3)	21 (22.6)	
Stage V plus VI	129 (69)	64 (68.1)	65 (66.9)	
hsCRP (mg/L)	4.85 ± 5.23	4.45 ± 4.99	5.28 ± 5.49	0.297
HA1c (%)	7.51 ± 1.57	7.57 ± 1.57	7.43 ± 1.59	0.659
Concomitant medications				
OHA alone	65 (37.8)	27 (31.8)	38 (43.7)	0.107
Insulin alone	55 (29.4)	30 (31.9)	25 (26.9)	0.450

ABI: ankle brachial index; BMI: body mass index; SBP: systolic blood pressure; CAD: coronary artery disease; CVA: cerebral vascular accident; DM: diabetes mellitus; ESRD: end-stage renal disease; EVT, endovascular therapy; hsCRP: high-sensitivity C-reactive protein; HA1c: hemoglobin A1c; OHA, oral hypoglycemic agent.

**Table 2:** Summary of TASC lesions, distribution of diseased vessels, and interventional characteristics.

TASC II classification	EVT group (*n* = 94)	Bypass group (*n* = 93)	*P* value
Aortoiliac lesions			
A	16 (17.0)	15 (16.3)	0.225
B	11 (11.7)	21 (22.8)	
C	13 (13.8)	11 (12.0)	
D	13 (13.8)	16 (17.4)	
Femoropopliteal lesions			
C	60 (63.8)	29 (31.2)	<0.001
D	34 (36.2)	64 (68.8)	
Infrapopliteal lesions			
B	40 (42.6)	42 (45.2)	0.238
C	40 (42.6)	30 (32.3)	
D	14 (14.9)	21 (22.6)	
Lesion length (cm)	18.5 ± 3.6 (15–29)	23.4 ± 5.2 (15–33.5)	<0.001
Number of distal runoffs	1.78 ± 0.72	1.96 ± 0.79	0.105
1 vessel	38 (40.4)	31 (33.3)	0.146
2 vessels	40 (42.6)	35 (37.6)	
3 vessels	16 (17.0)	27 (29.0)	
Stent implantation	94 (100)	0	
BTK intervention	94 (100)	0	
Bypass surgery	0	93 (100)	
AK FPB	0	41 (44.1)	
AK FPB plus tibial artery	0	17 (18.3)	
BK FPB	0	23 (24.7)	
BK FPB plus tibial artery		12 (12.9)	

AK, above the knee; BK, below the knee; DPA, dorsalis pedis artery; EVT, endovascular therapy; FPB, femoropopliteal bypass; PTA, posterior tibial artery; TASC, intersociety consensus for the management of peripheral arterial disease.

**Table 3:** Study endpoints associated with treatment methods.

Parameters	EVT group (*n* = 94)	Bypass group (*n* = 93)	*P* value
Successful intervention at one month	93 (100)	94 (100)	
Direct revascularization	43 (45.7)	47 (50.5)	0.512
Postintervention ABI			
One month	0.92 ± 0.1	0.95 ± 0.16	0.113
36 months	0.53 ± 0.16	0.72 ± 0.14	<0.001
Primary patency			
12 months	70 (74.5)	76 (81.7)	0.231
24 months	52 (55.3)	62 (66.7)	0.112
36 months	37 (39.4)	55 (59.1)	0.007
Secondary patency			
24 months	74 (78.7)	79 (84.9)	0.27
36 months	61 (64.9)	73 (78.5)	0.039
Male	25 (26.6)	31 (33.3)	0.315
AFSR at 36 months	64 (68.1)	71 (76.3)	0.208
LEA at 36 months	32 (34.0)	29 (31.2)	0.954
Major	8 (8.5)	8 (8.6)	
Minor	23 (24.5)	21 (22.6)	
24-month mortality	15 (16.0)	9 (9.7)	0.199
36-month mortality	27 (28.7)	18 (19.4)	0.134
12-month CAD events	15 (16.0)	17 (18.3)	0.673
24-month CAD events	28 (29.8)	26 (28.0)	0.782
36-month CAD events	30 (31.9)	29 (31.2)	0.914
24-month CVA events	5 (5.3)	10 (10.8)	0.171
36-month CVA events	10 (10.6)	11 (11.8)	0.797
Concomitant medications			
Cilostazol treatment			
≤3 months	31 (33.0)	23 (24.7)	0.213
>3 months	63 (67.0)	70 (75.3)	
Statin treatment	32 (34.0)	42 (45.2)	0.120
Antiplatelet therapy	93 (1001)	94 (100)	1.000

ABI, ankle brachial index; AFSR, amputation-free survival rate; CAD, coronary artery disease; CVA, cerebrovascular accident; EVT, endovascular therapy; LEA, lower-extremity amputation; MALE, major adverse limb event.

**Table 4:** Cox regression analysis of primary patency.

Variable	Univariate		^#^Multivariate	
	HR^*∗*^ (95% CI^†^)	*P* value	ATE	*P* value
Age (years)	1.02 (0.99–1.04)	0.208		
Gender (F/M)	1.01 (0.7–1.77)	0.662		
BMI	0.99 (0.94–1.04)	0.59		
Smoking (yes/no)	1.36 (0.89–2.08)	0.155		
Hyperlipidemia (yes/no)	0.97 (0.55–1.65)	0·902		
CAD (yes/no)	0.95 (0.63–1.42)	0.798		
Hypertension (yes/no)	1.06 (0.66–1.72)	0·799		
DM (yes/no)	1.26 (0.81–1.96)	0.306		
ESRD (yes/no)	1.06 (0.67–1.69)	0.805		
Rutherford classification (V plus VI/III plus IV)	1.46 (0.87–2.46)	0.153		
hsCRP (mg/L)	1.03 (0.99–1.67)	0.1	1.02 (0.98–1.0)	0.399
Diabetic neuropathy	2.03 (1.34–3.08)	0.001	0.94 (0.5–1.74)	0.824
Ischemia severity				
ABI	1.26 (0.78–2.02)	0.341		
Statin treatment (yes/no)	0.38 (0.24–0.62)	<0.001	0.54 (0.33–0.9)	0.017
Cilostazol treatment (>3 Ms/≤3 Ms)	0.33 (0.22–0.51)	<0.001	0.46 (0.3–0.72)	0.001
Insulin alone (yes/no)	2.12 (1.38–3.25)	0.001	1.6 (0.91–2.81)	0.106
Involved leg (L/R)	1.18 (0.82–1.7)	0.364		
Intervention (bypass/stenting)	0.61 (0.41–0.92)	0.019	0.8 (0.52–1.22)	0.292
DR/IR	0.34 (0.22–0.53)	<0.001	0.47 (0.29–0.74)	0.001

^#^Multivariate, weighted Cox regression with average treatment effect (ATE), ^*∗*^HR, hazard ratio; ^†^CI, confidence interval. ABI, ankle brachial index; BMI, body mass index; CAD, coronary artery disease; DM, diabetes mellitus; ESRD, end-stage renal disease; DR, direct revascularization; hsCRP: high-sensitivity C-reactive protein; HA1c: hemoglobin A1c; IR, indirect revascularization.

**Table 5:** Multivariate Cox regression analysis of primary patency in the two groups.

Variable	Multivariate (EVT group)		Multivariate (bypass group)	
	HR^*∗*^ (95% CI^†^)	*P* value	HR^*∗*^ (95% CI^†^)	*P* value
hsCRP (mg/L)	1.02 (0.54–1.92)	0.96	1.96 (0.77–5.0)	0.157
Neuropathy	0.83 (0.38–1.82)	0.648	0.89 (0.29–2.71)	0.832
Statin treatment (yes/no)	0.74 (0.38–1.45)	0.381	0.59 (0.25–1.42)	0.241
Cilostazol treatment (>3 Ms/≤3 Ms)	0.46 (0.26–0.82)	0.009	0.33 (0.15–0.75)	0.008
Insulin alone (yes/no)	0.98 (0.46–2.09)	0.966	2.72 (0.94–7.87)	0.065
DR/IR	0.43 (0.22–0.81)	0.009	0.5 (0.2–1.23)	0.132

^*∗*^HR, hazard ratio; ^†^CI, confidence interval; DR, direct revascularization; EVT, endovascular therapy; hsCRP: high-sensitivity C-reactive protein; HA1c: hemoglobin A1c; IR, indirect revascularization.

## Data Availability

The data used to support the findings of this study are available from the corresponding author upon request after the author gets approval from the ethics committee.

## References

[1] Rodrigues B. T., Vangaveti V. N., Malabu U. H. (2016). Prevalence and risk factors for diabetic lower limb amputation: a clinic-based case control study. *Journal of Diabetes Research*.

[2] Spreen M. I., Gremmels H., Teraa M. (2016). Diabetes is associated with decreased limb survival in patients with critical limb ischemia: pooled data from two randomized controlled trials. *Diabetes Care*.

[3] Norgren L., Hiatt W. R., Dormandy J. A. (2007). Inter-society consensus for the management of peripheral arterial disease (TASC II). *European Journal of Vascular and Endovascular Surgery*.

[4] Hong M. S., Beck A. W., Nelson P. R. (2011). Emerging national trends in the management and outcomes of lower extremity peripheral arterial disease. *Annals of Vascular Surgery*.

[5] Jones W. S., Mi X., Qualls L. G. (2015). Trends in settings for peripheral vascular intervention and the effect of changes in the outpatient prospective payment system. *Journal of the American College of Cardiology*.

[6] Dormandy J. A., Rutherford R. B. (2000). Management of peripheral arterial disease (PAD). *Journal of Vascular Surgery*.

[7] Brosi P., Dick F., Do D. D., Schmidli J., Baumgartner I., Diehm N. (2007). Revascularization for chronic critical lower limb ischemia in octogenarians is worthwhile. *Journal of Vascular Surgery*.

[8] Iida O., Soga Y., Hirano K. (2012). Long-term results of direct and indirect endovascular revascularization based on the angiosome concept in patients with critical limb ischemia presenting with isolated below-the-knee lesions. *Journal of Vascular Surgery*.

[9] Jongsma H., Bekken J. A., Akkersdijk G. P., Hoeks S. E., Verhagen H. J., Fioole B. (2017). Angiosome-directed revascularization in patients with critical limb ischemia. *Journal of Vascular Surgery*.

[10] Lejay A., Georg Y., Tartaglia E. (2014). Long-term outcomes of direct and indirect below-the-knee open revascularization based on the angiosome concept in diabetic patients with critical limb ischemia. *Annals of Vascular Surgery*.

[11] Patel M. R., Conte M. S., Cutlip D. E. (2015). Evaluation and treatment of patients with lower extremity peripheral artery disease. *Journal of the American College of Cardiology*.

[12] Mills J. L., Conte M. S., Armstrong D. G. (2014). The society for vascular surgery lower extremity threatened limb classification system: risk stratification based on wound, ischemia, and foot infection (WIfI). *Journal of Vascular Surgery*.

[13] Stürmer T., Wyss R., Glynn R. J., Brookhart M. A. (2014). Propensity scores for confounder adjustment when assessing the effects of medical interventions using nonexperimental study designs. *Journal of Internal Medicine*.

[14] Zhang Y., Gerdtham U. G., Rydell H., Jarl J. (2020). Quantifying the treatment effect of kidney transplantation relative to dialysis on survival time: new results based on propensity score weighting and longitudinal observational data from Sweden. *International Journal of Environmental Research and Public Health*.

[15] Lammer J., Zeller T., Hausegger K. A. (2013). Heparin-bonded covered stents versus bare-metal stents for complex femoropopliteal artery lesions: the randomized VIASTAR trial (Viabahn endoprosthesis with PROPATEN bioactive surface [VIA] versus bare nitinol stent in the treatment of long lesions in superficial femoral artery occlusive disease). *Journal of the American College of Cardiology*.

[16] Geraghty P. J., Mewissen M. W., Jaff M. R., Ansel G. M. (2013). Three-year results of the VIBRANT trial of VIABAHN endoprosthesis versus bare nitinol stent implantation for complex superficial femoral artery occlusive disease. *Journal of Vascular Surgery*.

[17] Lammer J., Zeller T., Hausegger K. A. (2015). Sustained benefit at 2 years for covered stents versus bare-metal stents in long SFA lesions: the VIASTAR trial. *CardioVascular and Interventional Radiology*.

[18] Iida O., Uematsu M., Soga Y. (2011). Timing of the restenosis following nitinol stenting in the superficial femoral artery and the factors associated with early and late restenoses. *Catheterization and Cardiovascular Interventions*.

[19] Van Belle E., Nikol S., Norgren L. (2011). Insights on the role of diabetes and geographic variation in patients with criticial limb ischaemia. *European Journal of Vascular and Endovascular Surgery*.

[20] Belch J., Hiatt W. R., Baumgartner I. (2011). Effect of fibroblast growth factor NV1FGF on amputation and death: a randomised placebo-controlled trial of gene therapy in critical limb ischaemia. *The Lancet*.

[21] Meltzer A. J., Evangelisti G., Graham A. R. (2014). Determinants of outcome after endovascular therapy for critical limb ischemia with tissue loss. *Annals of Vascular Surgery*.

[22] Kougias P., Chen A., Cagiannos C., Bechara C. F., Huynh T. T., Lin P. H. (2009). Subintimal placement of covered stent versus subintimal balloon angioplasty in the treatment of long-segment superficial femoral artery occlusion. *The American Journal of Surgery*.

[23] Dick P., Wallner H., Sabeti S. (2009). Balloon angioplasty versus stenting with nitinol stents in intermediate length superficial femoral artery lesions. *Catheterization and Cardiovascular Interventions*.

[24] Schillinger M., Sabeti S., Loewe C. (2006). Balloon angioplasty versus implantation of nitinol stents in the superficial femoral artery. *New England Journal of Medicine*.

[25] Laird J. R., Katzen B. T., Scheinert D. (2010). Nitinol stent implantation versus balloon angioplasty for lesions in the superficial femoral artery and proximal popliteal artery. *Circulation: Cardiovascular Interventions*.

[26] Scheinert D., Scheinert S., Sax J. (2005). Prevalence and clinical impact of stent fractures after femoropopliteal stenting. *Journal of the American College of Cardiology*.

[27] Pereira C. E., Albers M., Romiti M., Brochado-Neto F. C., Pereira C. A. B. (2006). Meta-analysis of femoropopliteal bypass grafts for lower extremity arterial insufficiency. *Journal of Vascular Surgery*.

[28] Adam D. J., Beard J. D., Cleveland T. (2005). Bypass versus angioplasty in severe ischaemia of the leg (BASIL): multicentre, randomised controlled trial. *Lancet (London, England)*.

[29] Michaels J. A. (1989). Choice of material for above-knee femoropopliteal bypass graft. *British Journal of Surgery*.

[30] Berglund J., Björck M., Elfström J., SWEDVASC Femoro-Popliteal Study Group (2005). Long-term results of above knee femoro-popliteal bypass depend on indication for surgery and graft-material. *European Journal of Vascular and Endovascular Surgery*.

[31] Maini B. S., Orr R. K., O’Mara P., Hendershott T. (1996). Outcomes and resource utilization in a managed care setting for lower extremity vein bypass grafts. *The American Journal of Surgery*.

[32] Raptis S., Miller J. H. (1995). Influence of a vein cuff on polytetrafluoroethylene grafts for primary femoropopliteal bypass. *British Journal of Surgery*.

[33] Gerhard-Herman M. D., Gornik H. L., Barrett C. (2017). 2016 AHA/ACC guideline on the management of patients with lower extremity peripheral artery disease: a report of the American College of Cardiology/American Heart Association Task Force on clinical practice guidelines. *Circulation*.

[34] Zuliani Mauro M. F., Mangione J. A., Costa J. R. (2017). Randomized angiographic and intravascular ultrasound comparison of dual-antiplatelet therapy vs. triple-antiplatelet therapy to reduce neointimal tissue proliferation in diabetic patients. *Journal of Invasive Cardiology*.

[35] Iida O., Nanto S., Uematsu M., Morozumi T., Kitakaze M., Nagata S. (2008). Cilostazol reduces restenosis after endovascular therapy in patients with femoropopliteal lesions. *Journal of Vascular Surgery*.

[36] Soga Y., Yokoi H., Kawasaki T. (2009). Efficacy of cilostazol after endovascular therapy for femoropopliteal artery disease in patients with intermittent claudication. *Journal of the American College of Cardiology*.

[37] Iida O., Yokoi H., Soga Y. (2013). Cilostazol reduces angiographic restenosis after endovascular therapy for femoropopliteal lesions in the sufficient treatment of peripheral intervention by cilostazol study. *Circulation*.

[38] Bangalore S., Singh A., Toklu B. (2014). Efficacy of cilostazol on platelet reactivity and cardiovascular outcomes in patients undergoing percutaneous coronary intervention: insights from a meta-analysis of randomised trials. *Open Heart*.

[39] Lee C.-Y., Wu T.-C., Lin S.-J. (2021). Effects of postoperative percutaneous coronary intervention, pharmacologic treatment, and predisposing factors on clinical outcomes in patients with and without type 2 diabetes along with critical limb ischemia. *Clinical Therapeutics*.

[40] Chen W.-J., Chen Y.-H., Lin K.-H., Hsuan Ting C., Yeh Y.-H. (2011). Cilostazol promotes vascular smooth muscles cell differentiation through the cAMP response element-binding protein-dependent pathway. *Arteriosclerosis, Thrombosis, and Vascular Biology*.

[41] Hayashi S., Morishita R., Matsushita H. (2000). Cyclic AMP inhibited proliferation of human aortic vascular smooth muscle cells, accompanied by induction of p53 and p21. *Hypertension*.

[42] Sanada F., Kanbara Y., Taniyama Y. (2016). Induction of angiogenesis by a type III phosphodiesterase inhibitor, cilostazol, through activation of peroxisome proliferator-activated receptor-*γ* and cAMP pathways in vascular cells. *Arteriosclerosis, Thrombosis, and Vascular Biology*.

[43] Stavroulakis K., Borowski M., Torsello G. (2017). Association between statin therapy and amputation-free survival in patients with critical limb ischemia in the CRITISCH registry. *Journal of Vascular Surgery*.

[44] Gerhard-Herman M. D., Gornik H. L., Barrett C. (2017). 2016 AHA/ACC guideline on the management of patients with lower extremity peripheral artery disease: executive summary: a report of the American College of Cardiology/American Heart Association Task Force on clinical practice guidelines. *Journal of the American College of Cardiology*.

